# Clinical significance of circulating anti-p53 antibodies in European patients with hepatocellular carcinoma

**DOI:** 10.1038/sj.bjc.6690095

**Published:** 1999-02

**Authors:** R Saffroy, J-C Lelong, D Azoulay, M Salvucci, M Reynes, H Bismuth, B Debuire, A Lemoine

**Affiliations:** Service de Biochimie, 14 avenue Paul Vaillant Couturier, Villejuif Cedex, 94804, France; UMR 217 CNRS, CEA Fontenay-aux-Roses, France; Centre hépatobiliaire, 14 avenue Paul Vaillant Couturier, 94804 Villejuif Cedex, France; Service d'Anatomie pathologique et Faculté de Médecine Paris-Sud, 14 avenue Paul Vaillant Couturier, Villejuif Cedex, 94804, France

**Keywords:** hepatocellular carcinoma, p53 antibodies, liver surgery

## Abstract

p53 alterations are considered to be predictive of poor prognosis in hepatocellular carcinoma (HCC) and may induce a humoral response. Anti-p53 serum antibodies were assessed by enzyme-linked immunosorbent assay (ELISA) using purified recombinant human p53 on 130 European HCC patients before treatment and during the clinical course of the disease. p53 immunohistochemistry was performed on tumours from the 52 patients who underwent surgery, and DNA sequencing analysis was initiated when circulating anti-p53 antibodies were detected. Nine (7%) HCC patients had anti-p53 serum antibodies before treatment. During a mean period of 30 months of follow-up, all the negative patients remained negative, even when recurrence was observed. Of the nine positive patients, eight were still positive 12–30 months after surgery. The presence of anti-p53 serum antibodies was correlated neither with mutation of the *p53* gene nor the serum alpha-fetoprotein levels and clinicopathological characterics of the tumours. However, a greater incidence of vascular invasion and accumulation of p53 protein were observed in the tumours of these patients (*P* < 0.03 and *P* < 0.01 respectively) as well as a better survival rate without recurrence (*P* = 0.05). In conclusion, as was recently shown in pancreatic cancer, anti-p53 serum antibodies may constitute a marker of relative ‘good prognosis’ in a subgroup of patients exhibiting one or several markers traditionally thought to be of bad prognosis. © 1999 Cancer Research Campaign


*British Journal of Cancer* (1999) **79**(3/4), 604–610
© 1999 Cancer Research Campaign
Article no. bjoc.1998.0095

### Clinical significance of circulating anti-p53 antibodies in European patients with hepatocellular carcinoma

**R Saffroy[1], J-C Lelong[2], D Azoulay[3], M Salvucci[1], M Reynes[4], H Bismuth[3], B Debuire[1] and A Lemoine[1]**

[1]Service de Biochimie, 14 avenue Paul Vaillant Couturier, 94804 Villejuif Cedex, France; [2]UMR 217 CNRS, CEA Fontenay-aux-Roses, France; [3]Centre hépatobiliaire, [4]Service d'Anatomie pathologique et Faculté de Médecine Paris-Sud, 14 avenue Paul Vaillant Couturier, 94804 Villejuif Cedex, France

**Summary** p53 alterations are considered to be predictive of poor prognosis in hepatocellular carcinoma (HCC) and may induce a humoral response. Anti-p53 serum antibodies were assessed by enzyme-linked immunosorbent assay (ELISA) using purified recombinant human p53 on 130 European HCC patients before treatment and during the clinical course of the disease. p53 immunohistochemistry was performed on tumours from the 52 patients who underwent surgery, and DNA sequencing analysis was initiated when circulating anti-p53 antibodies were detected. Nine (7%) HCC patients had anti-p53 serum antibodies before treatment. During a mean period of 30 months of follow-up, all the negative patients remained negative, even when recurrence was observed. Of the nine positive patients, eight were still positive 12–30 months after surgery. The presence of anti-p53 serum antibodies was correlated neither with mutation of the *p53* gene nor the serum alpha-fetoprotein levels and clinicopathological characterics of the tumours. However, a greater incidence of vascular invasion and accumulation of p53 protein were observed in the tumours of these patients ($P < 0.03$ and $P < 0.01$ respectively) as well as a better survival rate without recurrence ($P = 0.05$). In conclusion, as was recently shown in pancreatic cancer, anti-p53 serum antibodies may constitute a marker of relative 'good prognosis' in a subgroup of patients exhibiting one or several markers traditionally thought to be of bad prognosis.

**Keywords:** hepatocellular carcinoma; p53 antibodies; liver surgery

Considerable experimental evidence indicates that the *p53* tumour-suppressor gene is involved in hepatocellular carcinoma (HCC), with a variable incidence according to the geographical origin of the patients. Mutation of the *p53* gene and cellular accumulation (nuclear and/or cytoplasmic) of the mutated or wild-type p53 protein have been considered as factors of poor prognosis in different types of cancer (for reviews see Soussi et al, 1994; Zusman, 1995). Investigation of cellular p53 accumulation by immunohistochemical methods, and sequence analysis of the *p53* gene, require tumorous tissue obtained by fine needle biopsy or examination of the surgical piece, which is a limiting step.

More recently, serum antibodies reacting with the p53 protein have been detected in cancer patients with a variety of neoplasms (Schlichtholz et al, 1992; Angelopoulou et al, 1994, 1996; Volkmann et al, 1993; Houbiers et al, 1995; Lubin et al, 1995*a*, *b*; Bourhis et al, 1996; Coomber et al, 1996; Gadducci et al, 1996; Ryder et al, 1996; Wild et al, 1995). A prerequisite for a B-cell response seems to be the accumulation in the tumour of a mutant or, less frequently, a wild-type p53 protein. Anti-p53 serum antibodies have been associated with poor prognosis and advanced stage in breast (Schlichtholz et al, 1992; Angelopoulou et al, 1996), head and neck (Bourhis et al, 1996), colorectal (Houbiers et al, 1995; Coomber et al, 1996) and ovarian (Angelopoulou et al, 1996; Gadducci et al, 1996) cancers. However, serological assessment of anti-p53 antibodies seems also to allow detection of tumours at an early and therefore more treatable stage (Lubin et al, 1995*b*; Trivers et al, 1995, 1996). Moreover, this non-invasive test is adapted to the follow-up of cancer patients in clinical routine laboratories.

Two previous studies have reported an incidence of 2% and 25%, respectively, for the presence of anti-p53 serum antibodies in Western populations of HCC patients (Angelopoulou et al, 1994; Volkmann et al, 1993). In these studies, the presence of circulating anti-p53 antibodies appeared to be independent of alpha-fetoprotein (AFP) levels, a currently used but poorly reliable marker and predictor of HCC. More recently, another study from a Japanese group showed an incidence of 32% of positive samples and an association with a poor prognosis (Shiota et al, 1997). No information was available about the follow-up of the patients.

Three years ago, we initiated a prospective study to determine the interest of including the assessment of anti-p53 antibodies in the follow-up of HCC patients. We also investigated whether it correlated with clinicopathological variables and patient survival. p53 immunohistochemistry was performed when the tumours were resected and nucleotide sequence of the entire coding region of the *p53* gene was determined in patients exhibiting anti-p53 serum antibodies.

We report here the results of this study on 130 consecutive European HCC patients tested for the presence of anti-p53 serum antibodies before treatment and periodically during the clinical course of the disease for a mean period of 30 months.

*Received 25 February 1998*
*Revised 26 June 1998*
*Accepted 14 July 1998*

*Correspondence to:* A Lemoine, Service de Biochimie, Hôpital Paul Brousse, 14 avenue Paul Vaillant Couturier, 94804 Villejuif Cédex, France

### PATIENTS AND METHODS

### Study population

Anti-p53 serum antibodies detection was performed on 130 consecutive outpatients with confirmed HCC (30 women, 100 men, mean age 54.8 years, range 18–85) between October 1994

and December 1995. All the patients were assessed before treatment and 2–10 times during the course of the disease. All the assays were performed in duplicate.

Diagnosis of HCC was made by ultrasonography and computerized tomography and serum AFP, and confirmed, upon resection, by histological examination of the surgical piece. Size (maximal diameter of the tumour), number of nodules and total volume of the tumour were calculated using imaging and volumetric scanning techniques, including intraoperative ultrasound. The number and size of nodules, the grade according to Edmonson's classification, the presence of portal or hepatic vein invasion and the infiltration of the fibrous capsule were reported by the pathologist. During the follow-up of the patients, abdominal ultrasound and thoracic and abdominal volumetric scanning were periodically performed. Bone scintigraphy was prescribed when there was bone pain.

In 105 out of 130 patients, HCC had developed on a cirrhotic liver, 20 out of 130 had chronic hepatitis; in five patients, HCC had developed on a normal liver and no known hepatotropic viruses were found. HCC was of viral origin in 81 patients, and consequent upon excessive alcohol consumption in 16 patients. In 28 patients, viral and alcoholic aetiological factors were associated.

Seventy-eight patients had undergone liver surgery (49 partial hepatectomy and 29 total hepatectomy followed by orthotopic liver transplantation). One to three treatments by arterial chemoembolization using doxorubicin according to a protocol reported previously by us (Bismuth et al, 1992), were performed in 105 patients. All the patients were tested for presence of circulating anti-p53 antibodies at least once before beginning chemotherapy.

We also tested two control groups, including the sera from 40 healthy subjects and from 36 patients with cirrhosis. In the latter group, there were 30 men and six women (mean age 47 years). The diagnosis of cirrhosis was made on the basis of liver biopsy histology. HCC was excluded in the latter patients by abdominal ultrasonography, CT scan, and AFP measurement.

### Detection of serum anti-p53 antibodies

The detection of anti-p53 antibodies in patient sera was performed with a commercially available enzyme-linked immunosorbent assay (Anti-p53 ELISA, Pharmacell Paris, distributed by Immunotech, Marseille, France). The assay was based on an indirect technique using microtitre plates coated with recombinant wild-type human p53 protein or with a control well coated with the neutral antigen. This assay was performed according to the manufacturer's instructions with the following specifications: 1/100 diluted patient serum was added for 60 min at 20–25°C, with shaking, to microtitre wells coated with recombinant wild-type human p53 protein (to detect specific anti-p53 antibodies), or with a control protein (to detect non-specific interactions). After washing, goat anti-human IgG antibody conjugated with peroxidase was added for 60 min at 20–25°C with shaking. Finally, the substrate 3,3′,5,5′-tetramethylbenzidine (TMB) was added for 10 min. The enzymatic process was stopped by adding 2 N sulphuric acid. Light absorption was measured at 450 nm on a spectrophotometer (Dynatec, Paris, France). In this assay, non-specific background of each sample corresponded to the absorbance measured on wells coated with control protein.

Anti-p53 antibodies were considered positive in a sample for an index value [specific signal of the sample (p53 net absorbance –control protein net absorbance) /specific signal of the lower positive manufacturer's control serum] ≥ 1.1. Positive sera were checked at least twice in two independent experiments and the follow-up of a patient positive for anti-p53 antibodies was performed on the same plate.

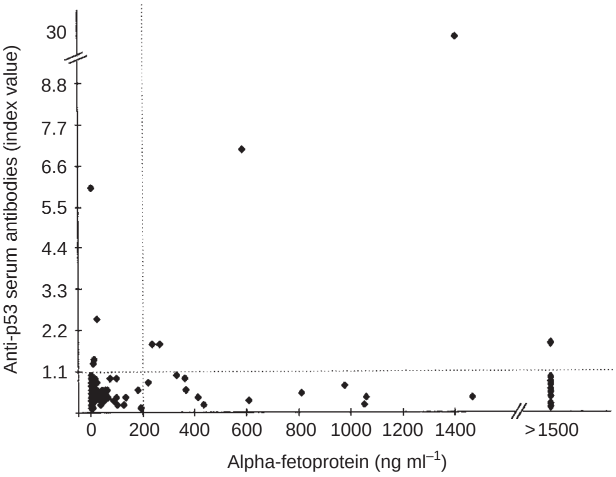

**Figure 1** Correlation between the index value of anti-p53 antibodies (positive threshold 1.1) and serum AFP levels

### Specificity of anti-p53 antibodies detection and purification of recombinant human p53

The validity of this test was established by the lack of anti-p53 antibodies detection in 200 sera from patients without malignancies (Lubin et al, 1995*a*), and in positive serum (dilution 1/100) of this study incubated for 30 min at 37°C with a range of concentrations of purified p53 protein (0.1–1 ng $ml^{-1}$) before titration. The purification of the p53 protein was performed according to the procedure that follows. The recombinant baculovirus used was vEV 55 p53 (O'Reilly and Miller, 1988), generously provided by Dr J Baudier. Nuclear extract of baculovirus-infected SF9 cells was first purified on a Q sepharose fast-flow column (Pharmacia, Uppsala, Sweden) as described previously (Delphin et al, 1994), then immunoaffinity purified on Protein A Sepharose Matrix covalently coupled to the monoclonal antibody Pab 122. Elution of p53 was carried out using a synthetic peptide comprising the Pab 122 epitope (amino acids 370–378). Eluted p53 was shown to be 95% pure on acrylamide gel (Lechner and Laimins, 1994) and stored in liquid nitrogen after extensive dialysis against 50% glycerol, 20 mM dithiothreitol and 1 mM Pefabloc [4-(2aminoethyl)-benzenesulphonylfluoride hydrochloride; Boerhinger, Mannheim, Germany] containing buffer.

### Immunohistochemical detection of p53 protein

Tissue sections were dewaxed in xylene, followed by graded alcohol, then rehydrated, microwaved in 0.01 M citrate buffer, pH 6.0, and blocked with normal horse serum. The monoclonal antibody Pab 1801 (Dako, Glostrup, Denmark), which recognizes a denaturation resistant epitope in the N-terminus of the molecule, was used to detect cellular accumulation of wild-type or mutant p53. Bound antibody was detected by incubation of the section with a biotinylated horse anti-(mouse Ig) antibody followed by streptavidin–horseradish peroxidase complex. Colour was developed with diaminobenzidine tetrahydrochloride and sections were counterstained with haemotoxylin before mounting. A negative

© Cancer Research Campaign 1999

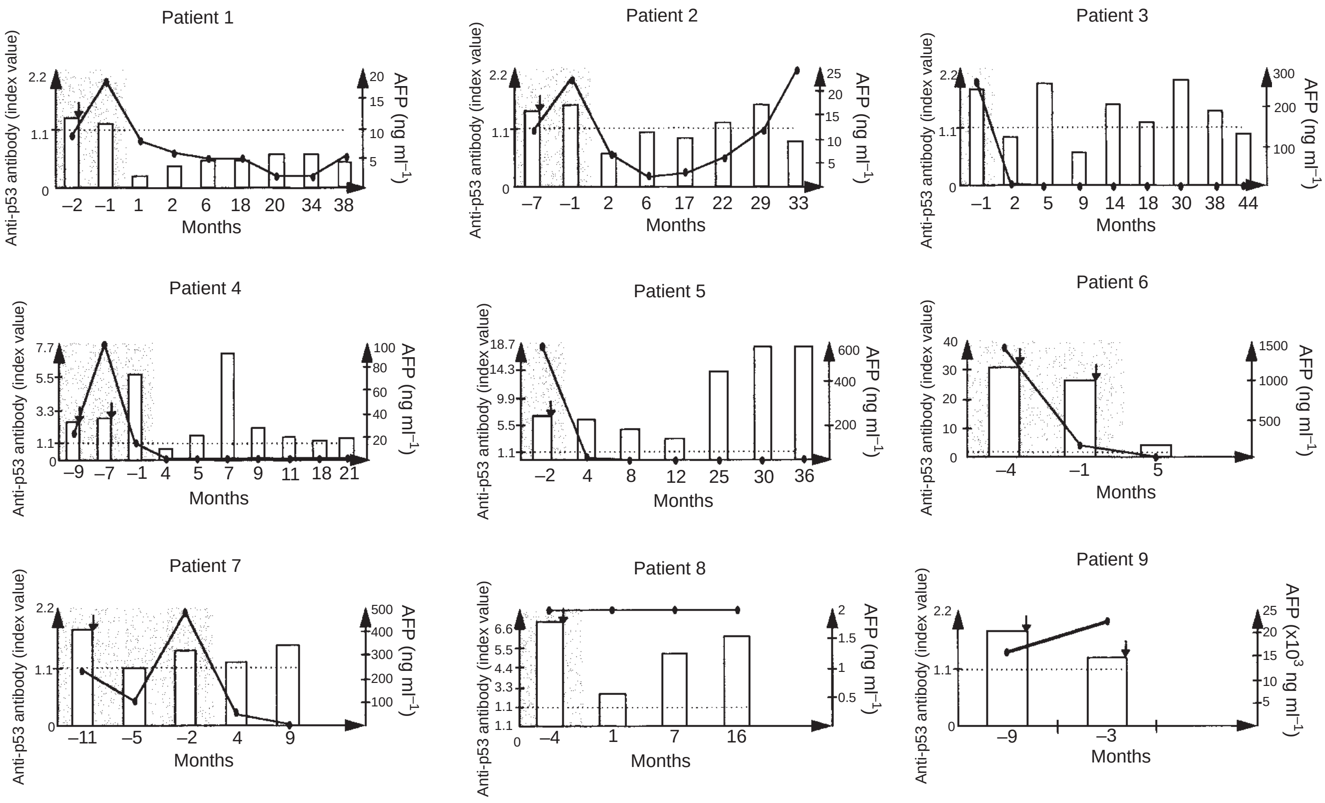

**Figure 2** Time course of anti-p53 serum antibodies in the nine patients seropositive for anti-p53 antibodies. The shaded area corresponds to the period preceding partial hepatectomy or orthotopic liver transplantation. Histograms represent the index value of anti-p53 antibodies (positive threshold 1.1). Curves represent serum AFP levels (normal < 20 ng ml$^{-1}$). ↓, Chemoembolization; ..., threshold control obtained by the replacement of primary antiserum with Tris buffer and a positive control (a positive case of human colon cancer) were included in each series. For each section, the percentage of tumour cells that stained positive for p53 was determined. Tumours were considered as positive when more than 20% of cells stained positively for p53.

### Tumour samples and DNA preparation

The tumour samples were dissected from surgical specimens and did not include any necrotic or non-cancerous liver tissue. They were then frozen immediately and stored in liquid nitrogen until use. In one case, tumour samples were obtained from paraffin sections of the fixed tumour. High molecular weight DNA was isolated by proteinase K and phenol/chloroform treatments, washed with 70% ethanol, vacuum dried, resuspended in water and quantified by optical densitometry and gel analysis.

### DNA sequence analysis

Tumour DNA from six of the nine ELISA p53 positive patients was available for DNA sequencing. Exons 2–11 were amplified in five independent polymerase chain reaction (PCR) amplifications, as previously reported by us (Bourdon et al, 1995), with one non-biotinylated primer and the other one biotinylated at the 5′ end. This allowed further purification of the PCR products on streptavidin-coated magnetic beads (Dynabeads M-280, streptavidin, Dynal, Oslo, Norway).

Sequencing was performed on both strands using dye terminator chemistry and ABI prism 310 automated DNA sequencer according to the manufacturer's recommendations (Perkin Elmer, Applied Biosystems, Courtaboeuf, France). Sequencing primers were as follows: exons 2–4, Ui1, Ui3, Li3A, Li4b; exons 5 and 6, Ui4B, Ui5, Li5, Li6B; exons 7–9, Ui6B, Ui7, Li7, Li9; exon 10, Ui9, Li10A; exon 11, Ui10, Li11 (Bourdon et al, 1995). Mutations were confirmed at least twice on independent PCR amplification products.

### Statistical analysis

Statistical analysis was carried out with the SAS software system (SAS Institute, Cary, NO, USA) and differences were considered significant when the probability values obtained from the statistical test were 0.05 or less. Clinical and biological characteristics of patients were expressed as means ± s.d. Comparisons were performed with the $\chi^2$ Fischer exact test. Survival curves without recurrent disease were constructed using the actuarial Kaplan–Meier method. The statistical significance between curves was analysed by the log-rank test.

### RESULTS

### Incidence and specificity of p53 serum antibodies in HCC patients

We tested for anti-p53 serum antibodies in a series of 130 consecutive outpatients with confirmed HCC; 19 of them presented with extrahepatic metastases. Of these 130 patients, only nine (7%) had

 *© Cancer Research Campaign 1999*

**Table 1** Characteristics of the tumours according to the presence or absence of serum anti-p53 antibodies in patients who underwent surgery

| | Anti-p53 negative (*n* = 70) | Anti-p53 positive (*n* = 8) | *P*-value |
|---|---|---|---|
| Underlying liver | | | |
| Cirrhosis | 55 | 5 | ns |
| Chronic hepatitis | 4 | 0 | ns |
| No cirrhosis | 11 | 3 | ns |
| Aetiology | | | |
| Viral | 44 | 5 | ns |
| Alcoholic | 9 | 2 | ns |
| Viral + alcoholic factors | 15 | 0 | ns |
| No aetiological factor | 2 | 1 | ns |
| Number of nodules | | | |
| 1 | 33 | 4 | ns |
| 2 | 19 | 2 | ns |
| ≥ 3 | 18 | 2 | ns |
| Diameter of nodules | | | |
| < 3 cm | 31 | 2 | ns |
| ≥ 3 cm | 39 | 6 | ns |
| Capsule | | | |
| Present | 18 | 4 | ns |
| Absent of infiltrated | 52 | 4 | ns |
| Edmonson's grade | | | |
| I | 5 | 1 | ns |
| II | 27 | 5 | ns |
| III | 12 | 1 | ns |
| IV | 8 | 0 | ns |
| Vascular invasion | | | |
| Yes | 24 | 7 | |
| No | 28 | 1 | <0.03 |
| p53 immunohistochemistry | | | |
| Positive | 7 | 6 | |
| Negative | 45 | 1 | <0.01 |

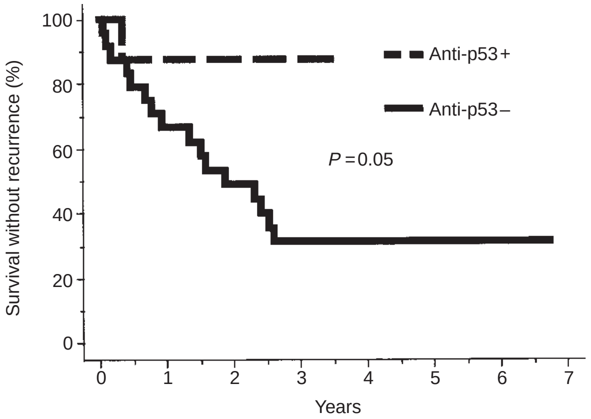

**Figure 3** Survival rate without recurrence in the eight patients positive for the presence of anti-p53 serum antibodies compared with the 24 patients who exhibited vascular invasion in their tumour, but no serum anti-p53 antibodies circulating anti-p53 antibodies before treatment (chemotherapy and/or surgery); one patient out of the nine positive patients for anti-p53 antibodies had extrahepatic metastases.

To check the specificity of the anti-p53 antibodies ELISA assay, we tested two control populations. The 40 healthy subjects assayed for circulating anti-p53 antibodies tested negative; two patients out of the 36 cirrhotic patients were found positive. We also incubated sera from the nine positive patients with increasing concentrations of purified p53 antigen to check whether the antibodies detected in the serum were well directed against the p53 protein. The ELISA was then performed and progressive disappearance of the signal was observed. Complete extinction of the signal was obtained for a concentration of 1 ng $ml^{-1}$ of p53 protein added to each of the nine serum samples tested (data not shown).

### Correlation between detection of circulating anti-p53 antibodies and AFP levels

The presence of anti-p53 antibodies in serum was not correlated with serum AFP levels. Indeed, of the 130 patients tested, 81 had serum AFP levels above the normal range (62%), whereas only nine patients had anti-p53 serum antibodies (Figure 1). However, it is interesting to note that, before surgery, one patient out of the nine positive patients for anti-p53 serum antibodies (patient 8 in Figure 2) had AFP levels in the normal range. In the post-operative period, AFP levels decreased to normal for eight of these nine patients, although seven of them still tested positive for anti-p53 serum antibodies.

### Biological course of the nine patients positive for anti-p53 serum antibodies

Several serum samples were obtained at different times during the course of the disease. All the patients who tested negative at diagnosis for anti-p53 circulating antibodies remained negative during the follow-up, besides variations in AFP levels. The follow-up of the nine positive patients is shown in Figure 2. Eight of them had received at least one treatment by arterial chemoembolization without subsequent disappearance of anti-p53 serum antibodies. Surgery was not possible for one of them (patient 9), who was treated by chemoembolization. He had developed lung and bone metastases associated with a considerable increase of AFP levels (14 125 to 22 440 ng $ml^{-1}$) and died during the course of the study. The other eight patients (patients 1–8) had undergone partial hepatectomy ($n = 4$) or complete hepatectomy followed by orthotopic liver transplantation ($n = 4$). They all tested positive for anti-p53 serum antibodies before surgery, with index values varying from 1.1 to 31.0. After surgery, only one patient (patient 1) showed complete disappearance of these antibodies within a period of 2 months. This patient had undergone major hepatectomy for a voluminous HCC (20 cm) with a surgical margin close to the tumour limits. Thirty-four months later, no circulating anti-p53 antibodies were detected and no recurrent disease was diagnosed.

Patients 2–4 showed temporary disappearance of anti-p53 serum antibodies which rose again 1–21 months after surgery, whereas at the same time serum AFP levels remained normal. However, patient 2 exhibited a small progressive increase in AFP levels associated with positivity of anti-p53 serum antibodies. In May 1998, imaging and volumetric scanning techniques showed no recurrence of the disease in these three patients 29, 38 and 18 months after surgery respectively.

Patients 5–8 remained positive for circulating anti-p53 antibodies after surgery whereas AFP levels became normal, although patient 8 had normal AFP levels before surgery. Patients 6 and 7 were lost for follow-up, whereas patients 5 and 8 had no recurrent disease 36 and 22 months after surgery respectively.

© Cancer Research Campaign 1999

### Tumour characteristics of the patients positive for presence of anti-p53 serum antibodies

Of the 130 patients, 78 underwent surgery. All the resected livers were analysed by the same pathologist who was unaware of the anti-p53 serum antibodies results. The histological characteristics of the tumours of patients free of circulating anti-p53 antibodies ($n = 70$) were compared with the p53 seropositive patients (eight out of nine patients, Table 1). The presence of anti-p53 antibodies cannot be explained by differences in the underlying liver disease (five cirrhosis, three non-cirrhotic tissue), aetiology (two alcoholic, five viral cirrhosis and one without viral or alcoholic factors) or number of nodules (four patients had one nodule, two patients had two nodules, and two patients had more than three nodules). The diameter of the main nodule varied from 2 to 20 cm. All the tumours were encapsulated, but in half of them the capsule was infiltrated. Edmonson's classification was analysed when the tumour was not completely necrosed and showed one type I, five type II and one type III.

Conversely, considerable incidence of portal or hepatic vein invasion was observed in the group of patients positive for anti-p53 serum antibodies (seven out of eight patients). This incidence was also analysed in the group of 70 patients operated for HCC and negative for anti-p53 antibodies. Only 52 out of 70 tumours were available for such an analysis because the remaining tumours showed necrosis. Of these 52 tumours, 24 exhibited vascular invasion. The difference between the two groups was significant ($P < 0.03$, $\chi^2$ Fischer exact test). We also compared the survival rate without recurrence between these two groups using the Kaplan–Meier technique. The log-rank analysis exhibited a significant difference in the duration of survival without recurrence between the two groups ($P = 0.05$; Figure 3) towards longer survival in the group of patients positive for anti-p53 serum antibodies.

### Association between the presence of anti-p53 serum antibodies and expression of p53 in the corresponding tumour p53 immunohistochemistry was performed on the tumours of operated patients when no necrosis was observed. Of the nine patients who had anti-p53 serum antibodies, seven were investigated. Six out of the seven tumours analysed exhibited an accumulation of p53 protein. Only seven of the 52 tumours from anti-p53 antibody-negative patients exhibited nuclear p53 accumulation ($P<0.01$, Table 1).

Sequence analysis of the entire coding region (exons 2–11) of the *p53* gene was performed on tumour DNAs from patients who had anti-p53 serum antibodies (one tumour was necrosed and one was not available for DNA preparation). Only one of the six samples analysed was mutated (exon 6, codon 195, Leu→Phe).

### DISCUSSION

We studied 130 consecutive patients with proven hepatocellular carcinoma and found a humoral response to p53 in nine of them (7%). Presence of anti-p53 serum antibodies significantly correlated with accumulation of p53 protein in the tumour, and was associated with vascular invasion. No significant correlation was found between the presence of anti-p53 serum antibodies and AFP levels, number and size of nodules, and prognosis. However, a significant higher survival rate without recurrence was observed in the group of patients positive for serum anti-p53 antibodies.

The test used in our study and derived from the assay developed by Lubin et al (1995*a*) allows great specificity. It has previously been used for assay on 1000 sera from cancer patients (breast, lung, colon, pancreas, thyroid, leukaemia), and on 200 sera from patients without malignancy. We also tested a series of 40 healthy patients; all gave negative results. In a population of 36 cirrhotic patients, two (5.5%) tested positive. It is possible that the cirrhotic liver of these seropositive patients harboured occult HCC-producing anti-p53 antibodies. Similarly, the presence of circulating anti-p53 serum antibodies has been observed in heavy smokers and bettel users (Lubin et al, 1995*b*; Kaur et al, 1997). In our assay, the use of a control well (serum of the patient without p53 antigen) for each test erases the non-specific reaction. Therefore, despite a great variation in the background of the signal from one serum to another, false-positive results, as could be the case with the serum of patients suffering from an autoimmune disease, can be avoided. In addition, the progressive and complete disappearance of the signal when positive sera are incubated with a range of increased concentrations of purified p53 protein proves the specificity of our assay, but it cannot be ruled out that a certain lack of sensitivity provides some false-negative results. Indeed, the incidence of 7% found in this study is higher than the 2% reported by Angelopoulou et al (1994), and lower than the 25% and 32% reported by Volkmann et al (1993) and Shiota et al (1997) respectively. These discrepancies can probably be explained by the different geographical origins of the tumours and by the nature of the assays used by the different groups. Angelopoulou et al (1994) used an ELISA with coated anti-p53 antibodies to avoid denaturation of the p53 protein, and reported an incidence of 2% for presence of anti-p53 serum antibodies in HCC. Volkmann et al (1993) developed an immunoblot assay, difficult to use routinely and probably the cause of false-positive results because of the presence of non-specific bands. This could account for the high incidence reported in their series. Shiota et al (1997) used an ELISA developed by Diannova (Munster, Germany). Recently, this test showed a lack of specificity in head and neck cancer with a control population exhibiting 24% of positivity (Wollenberg et al, 1997). The absence of a control well could explain these false-positive results. These previously reported high incidences of anti-p53 serum antibodies in liver tumours is different from what has been reported in other tumour types. Indeed, presence of anti-p53 serum antibodies is usually indicative of abnormal p53 protein expression in a high proportion of tumour cells; but the incidence of circulating anti-p53 antibodies is generally lower than the incidence of p53 protein accumulation in tumours (Lubin et al, 1995*a*). For example, in different series of breast and lung carcinomas, in which p53 immunohistochemistry analysis showed 20–40% and 60–70% positivity, respectively, the presence of anti-p53 serum antibodies was reported to be 15% and 30% respectively (Schlichtholz et al, 1992; Lubin et al, 1995*b*). In European HCC, we and others have previously reported p53 protein accumulation ranging from 31% to 45% (d'Errico et al, 1994; Volkmann et al, 1993; Bourdon et al, 1995), and *p53* gene mutation to be in the vicinity of 15% (Challen et al, 1992; Kress et al, 1992; Debuire et al, 1993). Thus, the 7% incidence of anti-p53 serum antibodies found in our series is in agreement with the level expected.

All the patients were tested before chemotherapy and/or surgery. There was no association between presence of anti-p53 serum antibodies and patient sex or age, clinical stage and histological type of the tumour, underlying disease, number and size of nodules or presence of extrahepatic metastases. These results are in agreement with the findings of Ryder et al (1996) and Volkmann et al

*© Cancer Research Campaign 1999*

(1993) but show a discrepancy with the results of Shiota et al (1997), who reported a correlation with the number of nodules. No correlation was observed with AFP levels, as previously reported by others (Volkmann et al, 1993; Raedle et al, 1995; Ryder et al, 1996). Indeed, circulating anti-p53 antibodies were detected in one out of nine patients with normal levels of AFP before surgery and in seven patients with normal levels of AFP after surgery.

Our data show that presence of circulating anti-p53 antibodies correlates with p53 protein overexpression in the tumour because seven out of eight patients positive in ELISA also tested positive in immunohistochemistry. This relationship between p53 overexpression in the tumour and presence of anti-p53 serum antibodies has already been reported in different types of malignancies including HCC (Davidoff et al, 1992; Angelopoulou et al, 1996; Ryder et al, 1996; Trivers et al, 1996). However, cellular accumulation of the p53 protein does not appear to be the sole contributing factor. Mutation of specific exons of the *p53* gene has also been incriminated. It has been postulated that the development of an immune response to a mutant protein may depend on mutant p53 protein complexes with a 70-kDa heat shock protein (HSP70) (Davidoff et al, 1992). However, this hypothesis has been questioned (Wild et al, 1995). In the present study, only one tumour out of six was found mutated, although complete sequencing of the *p53* gene was performed. This low incidence of mutations in the *p53* gene in HCC is in agreement with that which has been reported in the literature (Challen et al, 1992; Kress et al, 1992; Debuire et al, 1993). The accumulation of wild-type p53 protein can be explained by means of the formation of a complex with a cellular (ex: mdm2) or viral (ex: HBx) protein. We have shown that the tumours of all the patients with anti-p53 serum antibodies exhibited microveinule invasion associated with cellular accumulation of the p53 protein. This data raises the hypothesis that tumours preferentially elicit an immune response against p53 when a vascular invasion is observed. This suggests that the association of p53 accumulation with presentation of the antigen to the bloodstream seems to be required to induce a humoral response.

Presence of anti-p53 serum antibodies is regarded as a bad prognostic factor in breast and head and neck cancer, or as an increased risk for relapse but not death in ovarian and colon cancer (Schlichtholz et al, 1992; Houbiers et al, 1995; Angelopoulou et al, 1996; Bourhis et al, 1996; Coomber et al, 1996). As previously mentioned, eight patients of our series operated for HCC had anti-p53 serum antibodies, and at least one factor of bad prognosis as defined by the experience of different groups (Akashi et al, 1991; Yamashita et al, 1991; Bismuth et al, 1993; Ganne-Carié et al, 1996). However, they all had a survival rate without recurrence significantly higher than that of the 24 patients with vascular invasion but without circulating anti-p53 antibodies ($P = 0.05$, log-rank analysis). A similar observation has been reported in pancreatic tumours of advanced stage (Gansauge et al, 1996).

In conclusion, we observed a low incidence of circulating anti-p53 antibodies in a population of 130 European patients followed for proven HCC (9 out of 130, 7%). The humoral response did not seem to correlate with the clinicopathological features of the disease. However, the majority of patients who had anti-p53 serum antibodies exhibited cellular accumulation of p53 protein associated with microvascular invasion in their tumour compared with the group of seronegative patients. In most of them (five out of six), no mutation of the *p53* gene was detected. Presence of anti-p53 serum antibodies does not seem to be associated here with a poor prognosis. Further follow-up of these patients and analysis of a greater number of cases will probably lead us to consider whether anti-p53 serum antibodies constitute a marker of relative 'good prognosis' in a subgroup of patients exhibiting one or several markers traditionally thought to be of bad prognosis. This test could be add to the other tests such as serum AFP levels and radiographic scanning.

### ACKNOWLEDGEMENTS

This study was supported by grants from the Faculté de Médecine Paris-Sud. We thank Valérie Caillez for statistical analysis and Catherine Ouvrard for the help she provides us.

### REFERENCES

Akashi Y, Koreeda C, Enomoto S, Uchiyama S, Mizono T, Shiozaki Y, Sameshima Y and Inoue K (1991) Prognosis of unresectable hepatocellular carcinoma: an evaluation based on multivariate analysis of 90 cases. *Hepatology* **14**: 262–268

Angelopoulou K, Diamandis EP, Sutherland DJA, Kellen JA and Bunting PS (1994) Prevalence of serum antibodies against the p53 tumour suppressor gene protein in various cancers. *Int J Cancer* **58**: 480–487

Angelopoulou K, Rosen B, Stratis M, Yu H, Solomou M and Diamandis EP (1996) Circulating antibodies against p53 protein in patients with ovarian carcinoma. *Cancer* **15**: 2146–2152

Bismuth H, Morino M, Sherlock D, Castaing D, Miglietta C, Cauquil P and Roche A (1992) Primary treatment of hepatocellular carcinoma by arterial chemoembolization. *Am J Surg* **163**: 387–394

Bismuth H, Chiche L, Adam R, Castaing D, Diamond T and Dennison A (1993) Liver resection versus transplantation for hepatocellular carcinoma in cirrhotic patients. *Ann Surg* **218**: 145–151

Bourdon JC, D'Errico A, Paterlini P, Grigioni W, May E and Debuire B (1995) p53 protein accumulation in European hepatocellular carcinoma is not always dependent on p53 gene mutation. *Gastroenterology* **108**: 1176–1182

Bourhis J, Lubin R, Roche B, Koscielny S, Bosq J, Dubois I, Talbot M, Marandas P, Schwaab G, Wibault P, Luboinski B, Eschwege F and Soussi T (1996) Analysis of p53 serum antibodies in patients with head and neck squamous cell carcinomas. *J Natl Cancer Inst* **88**: 1228–1233

Challen C, Lunec J, Warren W, Collier J and Bassendine MF (1992) Analysis of the p53 tumour-suppressor gene in hepatocellular carcinomas from Britain. *Hepatology* **16**: 1362–1366

Coomber D, Hawkins NJ, Clark M, Meagher A and Ward RL (1996) Characterization and clinicopathological correlates of serum anti-p53 antibodies in breast and colon cancer. *J Cancer Res Clin Oncol* **122**: 757–762

Davidoff AM, Iglehart JD and Marks JR (1992) Immune response to p53/HSP70 complexes in breast cancers. *Proc Natl Acad Sci USA* **89**: 3439–3442

Debuire B, Paterlini P, Pontisso P, Basso G and May E (1993) Analysis of the p53 gene in European hepatocellular carcinomas and hepatoblastomas. *Oncogene* **8**: 2303–2306

Delphin, Cahen P, Lawrence JJ and Baudier J (1994) Characterization of baculovirus recombinant wild type p53. Dimerization of p53 is required for high affinity DNA binding and cysteine oxidation inhibits p53 DNA binding. *Eur J Biochem* **223**: 683–692

D'Errico A, Grigioni WF, Fiorentino M, Baccarini P, Grazi GL and Mancini AM (1994) Overexpression of p53 protein and Ki67 proliferative index in hepatocellular carcinoma. *Pathol Int* **44**: 682–687

Gadducci A, Ferdeghini M, Buttitta Fiama, Fanucchi A, Annicchiarico C, Prontera C, Bevilacqua G and Genazzani R (1996) Preoperative serum antibodies against the p53 protein in patients with ovarian endometrial cancer. *Anticancer Res* **16**: 3519–3524

Ganne-Carie N, Chastang C, Chapel F, Munz C, Pateron D, Sibony M, Deny P, Trinchet JC, Gallard P, Guettier C and Beaugrand M (1996) Predictive score for the development of hepatocellular carcinoma and additional value of liver large cell dysplasia in western patients with cirrhosis. *Hepatology* **23**: 1112–1118

Gansauge S, Gansauge F, Negri G, Galle P, Mûller J, Nûssler AK, Poch B and Beger HG (1996) The role of anti-p53 autoantibodies in pancreatic disorders. *Int J Pancreatol* **19**: 171–178

Houbiers JGA, Van Der Burg SH, Van De Watering LMG, Tollenaar RAEM, Brand A, Van De Velde CJH and Melief CMJ (1995) Antibodies against p53 are associated with poor prognosis of colorectal cancer. *Br J Cancer* **72**: 637–641

Kaur J, Srivastava A and Ralhan R (1997) Serum p53 antibodies in patients with oral lesions: correlation with p53/HSP70 complexes. *Int J Cancer* **74**: 609–613

Kress S, Jahn UR, Buchmann A, Bannasch P and Schwarz M (1992) p53 mutations in human hepatocellular carcinomas from Germany. *Cancer Res* **52**: 3220–3223

Lechner MS and Laimins CA (1994) Inhibition of p53 binding by human papillomavirus E6 proteins. *J Virol* **68**: 4262–4273

Lubin R, Schlichtholz B, Teillaud JL, Garay E, Bussel A, Wild CP and Soussi T, (1995*a*) p53 antibodies in patients with various types of cancer: assay, identification, and characterization. *Clin Cancer Res* **1**: 1463–1469

Lubin R, Zalcman G, Bouchet L, Tredaniel J, Legros Y, Cazals D, Hirsch A and Soussi T (1995*b*) Serum p53 antibodies as early markers of lung cancer. *Nature Med* **7**: 701–702

O'Reilly DR and Miller LK (1988) Expression and complex formation of Simian virus 40 large T antigen and mouse p53 in insect cell. *J Virol* **62**: 3109–3119

Raedle J, Roth WK, Oremek G, Caspary WF and Zeuzem S (1995) α-Fetoprotein and p53 autoantibodies in patients with chronic hepatitis C. *Dig Dis Sci* **40**: 2587–2594

Ryder SD, Rizzi PM, Volkmann M, Metivier E, Pereira LMM, Galle PR, Naoumov NV, Zentgraf and Williams R (1996) Use of specific Elisa for the detection of antibodies directed against p53 protein in patients with hepatocellular carcinoma. *J Clin Pathol* **49**: 295–299

Schlichtholz B, Legros Y, Gillet D, Gaillard C, Marty M, Lane D, Calvo F and Soussi T (1992) The immune response to p53 in breast cancer patients is directed against immunodominant epitopes unrelated to the mutational hot spot. *Cancer Res* **52**: 6380–6384

Shiota G, Kishimoto Y, Suyama A, Okubo M, Katayama S, Harada K, Ishida M, Hori K, Suou T and Kawasaki H (1997) Prognostic significance of serum anti-p53 antibody in patients with hepatocellular carcinoma. *J Hepatol* **27**: 661–668

Soussi T, Legros Y, Lubin R, Ory K and Schlichtholz B (1994) Multifactorial analysis of p53 alteration in human cancer: a review. *Int J Cancer* **57**: 1–9

Trivers GE, Cawley HL, Debenedetti VMG, Hollstein M, Marion MJ, Bennett WP, Hoover ML, Prives CC, Tamburro CC and Harris CC (1995) Anti-p53 antibodies in sera of workers occupationally exposed to vinyl chloride. *J Natl Cancer Inst* **87**: 1400–1407

Trivers GE, De Benedetti VMG, Cawley HL, Caron G, Harrington AM, Bennett WP, Jett JR, Colby TV, Tazelaar H, Pairolero P, Miller R and Harris C (1996) Anti-p53 antibodies in sera from patients with chronic obstructive pulmonary disease can predate a diagnosis of cancer. *Clin Cancer Res* **2**: 1767–1775

Volkmann M, Müller M, Hofmann WJ, Meyer M, Hagelstein J, Räth U, Kommerell B, Zentgraf H and Galle PR (1993) The humoral immune response to p53 in patients with hepatocellular carcinoma is specific for malignancy and independent of the a-fetoprotein status. *Hepatology* **18**: 559–565

Wild CP, Ridanpaa M, Anttila S, Lubin R, Soussi T, Husgafvel-Pursiainen K and Vainio H (1995) p53 antibodies in the sera of lung cancer patients: comparison with p53 mutation in the tumour tissue. *Int J Cancer* **64**: 176–181

Wollenberg B, Jan NV, Pitzke P, Reiter W and Stieber P (1997) Anti-p53 antibodies in serum of smokers and head and neck cancer patients. *Anticancer Res* **17**: 413–418

Yamashita Y, Takahashi M, Koga Y, Saito R, Nanakawa S, Katanaka Y, Sato N, Nakashima K, Urata J, Yoshizumi K, Ito K, Sumi S and Kan M (1991) Prognostic factors in the treatment of hepatocellular carcinoma with transcatheter arterial embolization and arterial infusion. *Cancer* **67**: 385–391

Zusman I (1995) The clinical relevance of p53 oncoprotein determination in cancer diagnosis and prognosis (review). *Oncology Rep* **2**: 143–150

*© Cancer Research Campaign 1999*
